# Effect of diversity on growth, mortality, and loss of resilience to extreme climate events in a tropical planted forest experiment

**DOI:** 10.1038/s41598-018-33670-x

**Published:** 2018-10-18

**Authors:** Chantal Hutchison, Dominique Gravel, Frédéric Guichard, Catherine Potvin

**Affiliations:** 1 Department of Biology, McGill University, Montreal, H3A 1B1 Canada; 2 Département de biologie, Université de Sherbrooke, Sherbrooke, J1K 2R1 Canada

**Keywords:** Climate-change ecology, Forest ecology, Tropical ecology

## Abstract

A pressing question is whether biodiversity can buffer ecosystem functioning against extreme climate events. However, biodiversity loss is expected to occur due to climate change with severe impacts to tropical forests. Using data from a ca. 15 year-old tropical planted forest, we construct models based on a bootstrapping procedure to measure growth and mortality among different species richness treatments in response to extreme climate events. In contrast to higher richness mixtures, in one-species plots we find growth is strongly regulated by climate events and we also find increasingly higher mortality during a consecutive three year dry event. Based on these results together with indicators of loss of resilience, we infer an effect of diversity on critical slowing down. Our work generates new methods, concepts, and applications for global change ecology and emphasises the need for research in the area of biodiversity-ecosystem functioning along environmental stress gradients.

## Introduction

Projections of future climate change include higher mean temperatures, water deficit intensity, and more intense El Niño-Southern Oscillation events^1^. How biological systems will respond to stress due to changes in temperature and moisture regimes is therefore paramount^2^. Forecasting the impact a changing climate will have on forest ecosystems is particularly important because they cover 30% of the land surface, support 80% of the world’s terrestrial biodiversity, and perform essential ecosystem functions and services such as carbon sequestration^3,4^. These impacts may include reduced growth and increases in mortality^5^. Several recent studies in forests have investigated the stability of ecosystem functioning, such as growth or productivity, in response to drought^6–13^ and suggest that there may be a positive link to diversity.

Here we define stability as the ability of a community to defy change (i.e. resistance and resilience)^14^. The basis for positive diversity-stability relationships is the insurance hypothesis. First proposed by^15^ and refined by^16–19^, the insurance hypothesis posits that species richness can act as a buffer against fluctuations in the environment. A key mechanism underlying insurance is species asynchrony, i.e. that species may independently have different responses to similar environmental conditions^20^. How these species respond to environmental conditions may be driven by biological traits^21^ as well as demography^22^. Furthermore, there may be more relevant aspects of diversity from which to discern diversity-stability relationships^23^. In a recent study^24^, the authors found empirical evidence for decreased stability in certain functions from an analysis of species abundances in five different functional groups (i.e. decomposition, carbon sequestration, pollination, pest control, and cultural values) which they posit is due to a weakened insurance capacity. They claim that there is a higher risk of failure in these functions under future environmental perturbations, although they do not consider perturbations of higher frequency and magnitude. Incidentally, stress, defined as extreme environmental conditions which are detrimental to ecosystem functioning^25^, is largely ignored, both empirically and theoretically, in biodiversity-ecosystem functioning (BEF) research^25,26^ although there are exceptions^27–32^.

Multiple mechanisms may drive BEF relationships during stressful events^33^. Niche complementarity predicts that heterogenous environments lead to higher mortality in monocultures than in mixtures because resources are used more efficiently in mixtures than in monocultures thus mediating the community-level response to stress intensity^34^. Complementary effects can also come from changes in the functional contribution of facilitative interactions among species. However, a stressful environment can also change the strength of per capita species interactions. Specifically, it is posited that the performance of more diverse communities improves relative to that of low diversity communities in stressful environments because of the emergence of interspecific facilitative interactions among individuals^35^. Both species-specific (complementarity) and per capita (stress gradient hypothesis) mechanisms suggest a stronger impact of stress events on monocultures than on mixture communities.

Detection of community-level stress response has been inferred from measures of resistance and resilience to stress. Pimm^36^ defined resistance as the magnitude of change in a response variable during a perturbation and resilience as how fast recovery occurs following a perturbation. Subsequently, there have been many different mathematical interpretations of these measures. In particular, resistance and resilience have been interpreted as “components” of temporal stability^37^ (and references therein). A recent study^38^ further proposes a bivariate framework for resilience in terms of normalized resistance and recovery metrics based on the undisturbed state in order to have a comparable measure of resilience between different systems. When introducing the concept of stress, including extreme climate events and tree stress response, individual variation becomes important and a new detection problem emerges. Therefore, separating the background variation in functioning from the signal due to stress means developing an approach to measure stability accounting for this variation.

The ability of trees to recover may be dampened by periods of stress such as drought or extreme heat^39,40^. This loss of resilience or critical slowing down can push forests to the boundary of massive die-off^41^. Recently it has been shown using satellite data from evergreen tropical forests in South America, Africa, and Asia/Australia that slowing down may be driven by low levels of precipitation (i.e. mean annual precipitation values which are less than 1,500 mm ⋅ yr^−1^)^42^. There are many indicators which may infer slowing down^43,44^. Such indicators are related to the resilience, and in particular the shape of the basin of attraction, of the current state. As environmental drivers push the current state to a tipping point, the slope of the basin of attraction decreases and, consequently, resilience to small perturbations decreases^23^. If one were to measure the temporal autocorrelation (TAC) at lag-1 of this state, it would approach 1 as consecutive states in time are closer to one another due to a flattened basin. However, lag-1 autocorrelation does not account for variation at higher lags; the power spectrum can show changes in the spectral properties of a time series before a transition. In other words, a power spectrum can determine how similar states are at higher lags by decomposing a time series into its component frequencies. Systems closer to a transition may exhibit spectral reddening (i.e. most of the variation in the series occurs at lower frequencies) because states tend to change slowly in time about this flattened basin. A particularly important question which, to the best of our knowledge, has not yet been explored in the literature is how diversity might impact slowing down. TAC and spectral reddening have the potential to provide novel insights to slowing down following extreme climate events.

Here we focus on the detection of stress response in forest communities. As our case study, we consider a long-running tropical tree diversity experiment^45^ and identify stressful events at the site from the standardised precipitation-evapotranspiration index^46^ (SPEI). We study how growth and mortality along diversity gradients are impacted by extreme climate events based on bootstrapping of tree data to generate the statistics need to account for potentially large individual variability. We hypothesize that differences in growth along a gradient of species richness should manifest mostly during wet or dry extreme climate events. In other words, monocultures should have greater variability in their growth compared to higher richness mixtures during stress. We also predict that mortality will be higher in monocultures during intense spells of drought as a result of lower resilience than higher diversity treatments. To determine whether the hypothesized mortality for monocultures may indicate sudden massive die-off we compute the temporal autocorrelation at lag-1 and the spectral density of growth time series. To study the relationship between species richness and slowing down, we compare these indicators across diversity treatments. Specifically we expect monocultures to exhibit spectral reddening whereas the power spectrum should become more flat at higher richness. Our approach improves our understanding and our ability to quantify stability under stress for forest ecosystems, and gives momentum to the study of the relationship between diversity and slowing down.

## Results

### Climate events identified from the SPEI

Based on a 21 year period from 1995 to 2016, we identify extreme climate events occurring approximately 10% of the time, moderate dry and wet events each occurring 25-30% of the time, and normal conditions persisting 35%. Normal years are identified based on the climate data (i.e. rainfall and evapotranspiration) over a 21 year period; because of the way SPEI is computed there is an unavoidable shifting baseline which depends on the period chosen. Climate extremes defined only using data from the early (or late) 1900s, would probably have identified more (or fewer) extreme climate events^29^.

**Figure 1:**
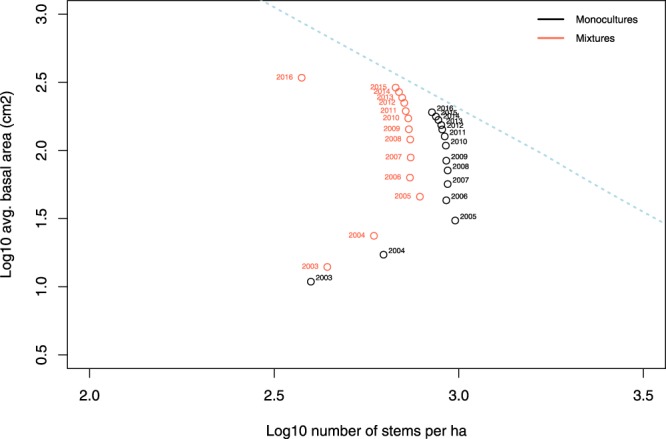
Development phase for the even-aged stands at Sardinilla. Two trajectories are shown for monocultures (black) and mixtures (red). As plot basal area increases the number of trees per hectare decreases over time starting in 2006, and the trajectory moves upwards and to the left as it converges to the self-thinning line with slope −3/2 (blue dotted line).

We chose the timeframe from 2006 to 2016 to construct our models and test our central hypotheses in order to control for stand development (Fig. 1). We see that from 2003 to 2005, the plots are in the establishment phase during which juveniles recruit into the size class in which diameter at breast height (DBH) can be measured (1.3 m). By 2015, plots are just reaching the self-thinning line of slope −3/2. Along this line, mortality is mostly driven by strong competition between stems. By 2006, plots in both monocultures and mixtures are out of the early development phase and on a trajectory towards self-thinning, but have not reached it. Therefore, this appears to be the period over which climate may be the main driver of growth and mortality^47^.

**Figure 2:**
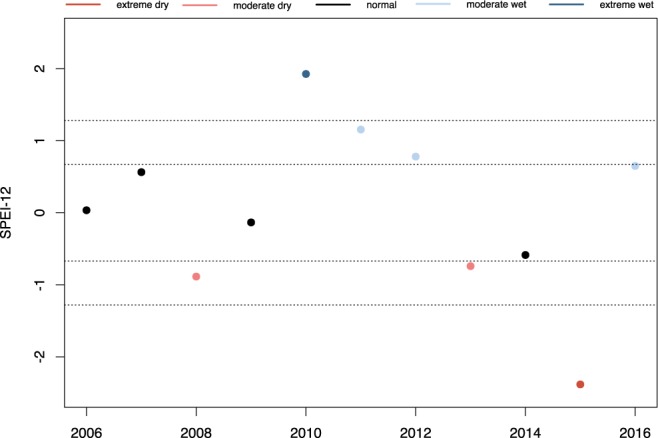
SPEI-12 (SPEI aggregated over twelve months) from December over 2006-2012. There is an extreme wet event in 2010 and a dry spell from 2013 to 2015.

In this timeframe, we find an extreme La Niña year in 2010 followed by two moderately wet years. Subsequently there is a series of dry years: 2013 and 2014 are moderately dry, and in 2015 there is a strong El Niño year with very dry conditions (Fig. 2).

### Growth and mortality models

**Figure 3:**
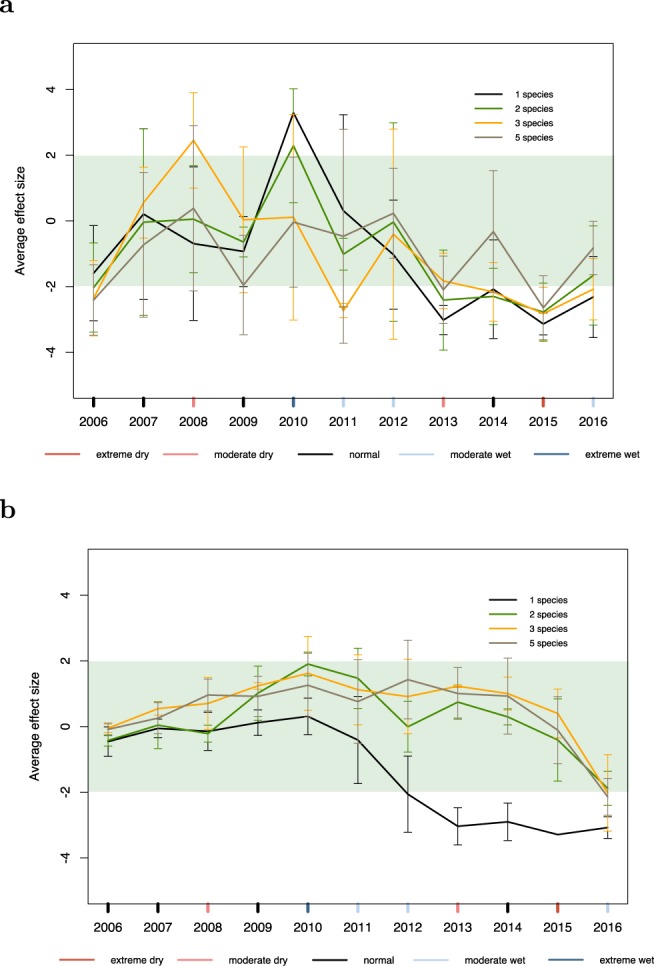
(**a**) Growth model and (**b**) mortality model. Average effect size (AES) over plots in monoculture, two-species mixtures, three-species mixtures, and five-species mixtures through time are shown. The error bars correspond to the mean ± the standard deviation for each year. SPEI-12 for a given year is indicated by the colour of the tick marks on the *x*-axis: normal is black, moderate wet is grey, moderate dry is coral, extreme wet is dark blue, and extreme dry is red. We find a clear distinction between growth and mortality between monocultures and higher species richness treatments compared to their null expectation which is represented by the honeydew band between [−2.0, 2.0].

We built null models of growth and mortality assuming a time-independent distribution which would be the case if extreme climate events were not driving inter-annual variations in growth or mortality at a given diversity level (see Methods). The growth model shows that in 2010, the extremely wet year, monocultures grew significantly better than expected by the null hypothesis (Fig. 3a). In plots with species richness, the effect of the 2010 wet year is less pronounced; three- and five-species mixtures do no better than expected at random. During the drought episode from 2013 through to 2015, we see that although all mixtures experience a decrease in growth, monocultures experience the largest decrease and five-species mixtures the smallest decrease (Fig. 3a). In the mortality model, we see that the number of dead trees is pushed further from the null expectation for monocultures during the continuous drought period from 2013 to 2015 (Fig. 3b). Mixtures show no significant difference from the null expectation in mortality during this period.

### Indicators of critical slowing down

**Figure 4:**
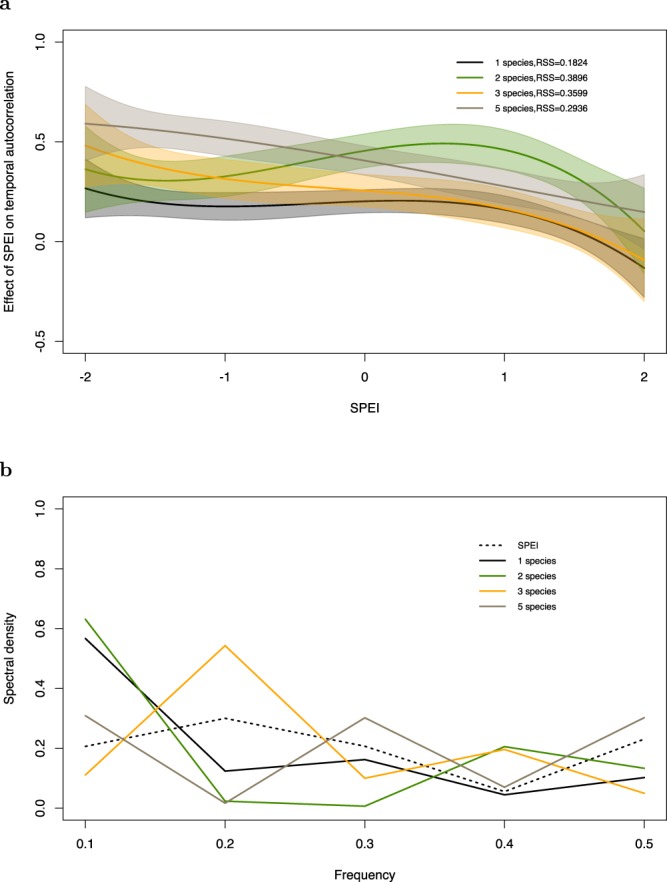
Indicators of critical slowing down. (**a**) Temporal autocorrelation at lag-1 of basal area increment time-series averaged over plots of the same species richness and (**b**) power spectrum of basal area increment time series averaged over plots of the same species richness and SPEI. We find that there is not a clear distinction between different species richness treatments and an increase in autocorrelation for dry conditions (i.e. increasingly negative SPEI). Monocultures and two-species mixtures have most of their variance in the lower frequency spectrum. This variance shifts to the right or to higher frequencies as richness increases.

The effect of SPEI on TAC at lag-1 shows a positive trend as SPEI decreases for all diversity treatments with confidence bands overlapping (Fig. 4a). Consequently, the strongest effect of SPEI on TAC is seen during dry relative to wet years. The power spectrum (Fig. 4b) indicates that both the monocultures and two-species plots have most of their variance in tree growth at lower frequencies, while the three-species plots have their variance at intermediate frequencies, and the five-species treatments have their variance spread over all frequencies. Furthermore, the monoculture spectrum seems to closely follow the spectrum computed for the SPEI.

## Discussion

Because the concepts of resistance and resilience as proposed by Pimm^36^ are very general, their expressions may be context dependent. For example, new standardised indices of resistance and resilience have been introduced to handle wet-dry cycles that are able to determine differences in stability between contrasting soils^48^. The effect sizes generated by our growth model are very similar to measures of resistance; however, our measure is adapted to studying stability in trees because we account for the large individual variation by adopting a bootstrapping procedure to compute both means and variances. Our results show strong indications of a positive diversity-stability relationship through a species richness effect on growth/mortality response to stress. This suggests that background variation may be especially relevant when considering how stress affects stability of ecosystem functioning in forest ecosystems contributing to BEF literature.

Taken together, the results from the mortality model, temporal autocorrelation, and the power spectrum suggest that monocultures may exhibit greater sensitivity to extreme conditions than mixtures. We found that during the successive dry years from 2013 to 2015 there was increasingly higher mortality in monocultures compared to mixtures, which suggests that intraspecific competition is greater than interspecific competition during extreme climate events. Furthermore, in monocultures we found an increase in temporal autocorrelation as conditions become drier as well as most of its variation in tree growth in lower frequencies. These results suggest that the die-off present in monocultures may be indicative of slowing down, or a loss of resilience. In particular, the comparison of the spectral density of monocultures to SPEI suggests that they are not simply mirroring the environment. Our results and methods should facilitate the inference of a relationship between diversity and slowing down from experiments with a longer time series and with more extreme stresses. For example, massive mortality in some tropical forests have be observed for precipitation values less than 1,500 mm ⋅ yr^−1 ^^42^. However, the precipitation at our site was never less than 1,900 mm ⋅ yr^−1^. This suggests that perhaps there exists a threshold at which a distinction in slowing down between diversity treatments can be found under more extreme stress.

We found that monocultures grow less than mixtures during a dry period from 2013 to 2015, culminating in 2015. Furthermore, mixtures experience less mortality than monocultures during these dry conditions. Higher growth and lower mortality in mixtures compared to monocultures is indicative of insurance effects^45^. Species complementarity in water uptake strategies is a possible mechanism for stress buffering in mixtures^11^. In fact, a series of studies conducted in Sardinilla between July 2007 and August 2008 found that complementary water uptake strategies between species was most pronounced during the dry season^49–51^. One of these studies^49^ also proposed that one of the deep soil water exploiters, *Tabebuia rosea*, may facilitate other species through hydraulic redistribution of water resources. The stress gradient hypothesis also predicts that per capita changes in species interactions under stressful conditions can explain the stress buffering effect of mixtures^33^. However, we currently have no theory predicting the relative importance of complementarity and per capita interactions for stress buffering. Testing such predictions would most likely require that empirical BEF studies include the measurement of individual interaction strength during stress events^52^.

Mortality in five-species mixtures is significantly different from mixtures with intermediate levels of species richness during the drought period from 2013 to 2015, although both experienced less mortality than monocultures. This difference between intermediate diversity levels and five-species mixtures suggests that there may be a strong dependence of species composition on mortality, which has been demonstrated previously in tropical forests^53^. This explanation seems likely because all the two- and three-species mixtures have different compositions while all five-species plots have the same species composition. The relative importance of richness and composition could be explored further, for example, by considering functional diversity^54^ of traits related to water uptake and soil nutrients and may help to clarify the relationship between diversity, mortality, and stability under stress in forests.

As more data becomes available from tree BEF experiments^55^, we anticipate our work will set a benchmark to determine the impact of climate change on planted forests worldwide as our detection procedure is sensitive to the variation of individual trees. Furthermore our measure is based on standardised measures enabling comparisons of stability between sites in different forest types and climates can be made. Our study has shown that species richness buffers growth in response to strong drought episodes in agreement with theory on the relationship between abiotic stress, complementarity, and per capita interactions. We found that there was higher mortality in monocultures than mixtures, which increases when subjected to higher stress intensity. By accounting for mortality in the context of stability, we found evidence for slowing down in monocultures and posited a diversity-slowing down relationship. More mechanistic investigations are required to fully disentangle the relationship between stress and BEF. We anticipate that our work will add to the growing literature on BEF across environmental stress gradients, which have application across many ecological systems.

## Methods

### Study site and experimental design

**Figure 5:**
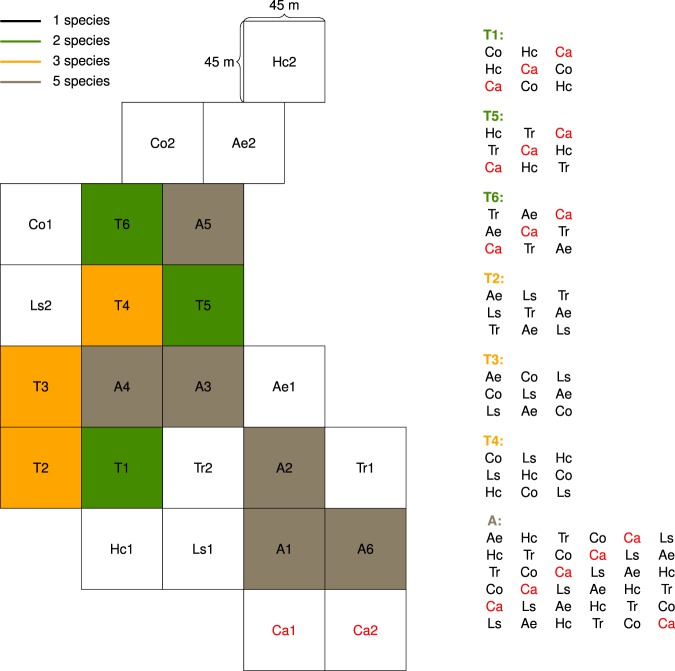
Schematic map of the Sardinilla planted forest. Diversity-levels and species neighbourhoods are shown. The species *Cordia alliodora* (Ca), which died in monoculture and in mixture, is excluded from this study and is indicated in red. The five remaining species are: *Anacardium excelsum* (Ae), *Cedrela odorata* (Co), *Hura crepitans* (Hc), *Luehea seemanni* (Ls), and *Tabebuia rosea* (Tr).

Our case study is a ca. 15 year-old tropical planted forest in Sardinilla, Central Panama (9° 19′N, 79° 38′W) (Fig. 5). It was established in 2001 as part of a network of tree biodiversity-ecosystem functioning (BEF) experiments called TreeDivNet (http://www.treedivnet.ugent.be/). A total of 5,566 tree seedlings <6-months old were planted in 24 contiguous 45 m by 45 m plots in a pasture of ~5 ha with mean 231.9 ± 8.93 seedlings per plot. Planting distances between individuals was 3 m following standard practices of reforestation in Panama. The planted forest has a substitutive randomised block design with species richness (monoculture, three-species, and six-species) and species combinations randomly allocated to allow quantification of biodiversity effects^56^. Six native tree species are represented based on their range of relative growth rates^57–59^. The fastest growing species are *Luehea seemanni* [Triana & Planch, Tiliaceae] and *Cordia alliodora* [(Ruiz & Pavon) Oken, Boraginaceae], the intermediate growing species are *Anacardium excelsum* [(Bertero & Balb. ex Kunth) Skeels, Anacardiaceae] and *Hura crepitans* [Linné, Euphorbiaceae], and the slow growing species are *Cedrela odorata* [Liné, Meliaceae] and *Tabebuia rosea* [(Bertol.) DC, Bignoniaceae]. *C. alliodora* suffered massive mortality in the monocultures and mixtures probably due to undrained and compact soil^60^. For this reason we do not include *C. alliodora* in our experiment. Consequently, we make comparisons using the actual diversity. At planting, there were 12 monocultures plots (two replicates of each of six species), 6 three-species plots, and 6 six-species plots. Using actual diversity removing *C. alliodora*, there are 10 monoculture plots (two replicates of each of five-species), 3 two-species plots, 3 three-species plots, and 6 five-species plots. The five-species plots were designed to have the same neighbourhoods whereas the two- and three-species plots all have different species compositions from each other. Undergrowth was manually cleared in the plantation three times per year to avoid competition with herbaceous vegetation. Measurements of tree height, diameter at breast height (~1.4 m) or DBH, and basal diameter (10 cm from the ground) were made every year at the onset of the dry season (end of December, early January) starting in 2001.

### Characterizing climate extremes

The region has a pronounced dry season from January to March^61^. Long-term meteorological data is available from nearby (≈30 km) Barro Colorado Island (BCI) research station^62^. It has a humid tropical climate^63^ with 25–50 mm per month during the dry season (January-March) and 250 mm per month wet season (May-November)^64^. The annual mean daily temperature is 33.1 °C and minimum is 21.7 °C^64^. We classify a year as being extremely dry/wet event, moderately dry/wet event or normal event by constructing the 12 month standardised precipitation-evapotranspiration index (SPEI-12^46^) from rainfall and potential evapotranspiration data at BCI. SPEI has been used as a drought index to study BEF in many studies (e.g^12,29^.). Unlike the standardised precipitation index (SPI^65^) the SPEI includes the effects of temperature; higher daily minimum temperature can increase water losses through evapotranspiration^66^. The SPEI is constructed as follows^46^. The *D*-series for month *i* is the difference between the precipitation, *P*_*i*_, and potential evapotranspiration, *PET*_*i*_: *D*_*i*_ = *P*_*i*_ − *PET*_*i*_. *D*_*i*_’s can be aggregated at different timescales, i.e. one month, three months, six months, twelve months, etc. Selection of the most appropriate distribution to model the *D*-series was based on extreme values and found to be described by a log-logistic distribution, *F*(*x*). Then, the SPEI is given by the standardized values of *F*(*x*). The classical approximation is given by^67^

1 $$SPEI=W-\frac{{C}_{0}+{C}_{1}W+{C}_{2}{W}^{2}}{1+{d}_{1}W+{d}_{2}{W}^{2}+{d}_{3}{W}^{3}}$$

where $W=\sqrt{-2\,{\rm{l}}{\rm{n}}(P)}$ for *P* ≤ 0.5 such that *P* = 1 − *F*(*x*) is the probability of being greater than a given value of *D*. Note that if *P* > 0.5, *P* becomes 1 − *P* and the sign of the SPEI is reversed. The constants are given by *C*_0_ = 2.515517, *C*_1_ = 0.802853, *C*_2_ = 0.010328, *d*_1_ = 1.432788, *d*_2_ = 0.189269, and *d*_3_ = 0.001308. For our study, we chose to look at the *D*-series aggregated over twelve months, i.e. a yearly water balance. This is because it coincided with the yearly growth and mortality measurements at Sardinilla from which we can infer the relationship between climate and tree data.

### Indicator of plot-level performance

Because tree growth and mortality are determinants of forest population dynamics^60,68^, they can be used to characterise its ecosystem state in response to extreme climate events when recruitment is controlled for. For each measurement period *t* from 2001 to 2017, we use DBH of each tree to construct its stem basal area increment and then sum all trees in a plot *P*. This basal area growth rate for plot *P* at time *t*, *BAGR*_*P*,*t*_ (in cm²), is an indicator of plot-level performance:

2 $${{\rm{B}}{\rm{A}}{\rm{G}}{\rm{R}}}_{P,t}=\frac{1}{\Delta t}\mathop{\sum }\limits_{i=1}^{{\rm{N}}{\rm{o}}.\,{\rm{t}}{\rm{r}}{\rm{e}}{\rm{e}}{\rm{s}}\,{\rm{i}}{\rm{n}}\,{\rm{p}}{\rm{l}}{\rm{o}}{\rm{t}}\,{\rm{P}}\,}\,\mathop{\sum }\limits_{j=1}^{{\rm{N}}{\rm{o}}.\,{\rm{o}}{\rm{f}}\,{\rm{s}}{\rm{t}}{\rm{e}}{\rm{m}}{\rm{s}}}(\frac{\pi {{\rm{D}}{\rm{B}}{\rm{H}}}_{P,i,j,t}^{2}}{4}-\frac{\pi {{\rm{D}}{\rm{B}}{\rm{H}}}_{P,i,j,t-\Delta t}^{2}}{4})$$

where Δ*t* is the number of days between measurements, one sum is over the number of trees in a plot, and the other sum is over the number of stems of a tree is the plot. By performance, we mean that two biological processes act to change BAGR_*P*,*t*_: growth and mortality^60^. In this case, *the first sum is done over both alive and dead trees*. If a tree has died we set the DBH at time *t* equal to the DBH at time *t* − Δ*t*, so mortality acts by adding zero to BAGR_*P*,*t*_ for a dead tree. Recruitment can also change BAGR_*P*,*t*_; however, undergrowth (including seedlings) are manually cleared three times per year at the site so recruitment is assumed not to contribute to performance^47^. found that BAGR at the plot-level were positively and significantly correlated with climate, in particular rainfall and temperature, for permanent forest plots in a Neotropical lowland Bolivia. Therefore, we expect that BAGR is an appropriate measure to use in order to study how performance changes across diversity-levels and climate events.

### Models of growth and mortality

We hypothesized that differences in growth and mortality along a gradient of species richness should manifest mostly during extreme climate events. To test this hypothesis we construct two different null models: a growth model and a mortality model, which represent our null expectation for each diversity treatment if climate is not driving year to year differences in performance. For each null model, we use R statistical software version 3.4.3 (2017-11-30) to generate 1000 boostrap samples for each plot by randomly sampling, with replacement, alive and dead trees with appropriate survival probabilities whilst preserving species identity in a given plot^60^. For the growth model, the pool is made of basal area increments for each tree in a plot over all years and we select a living tree with a probability of survival calculated for that year. We use a pool of increments from all years as a conservative choice which does not presuppose a relationship between plot-level growth and climate (Fig. S1). For the mortality model, the pool is made up of the basal area increments for the trees present in a given plot in a given year and we select a tree with a probability of survival which is constant year on year. Edge trees are removed from our analysis with the reasoning that those trees likely do not benefit from any full biodiversity effect. An effect size is computed to compare actual growth and mortality compared to the null expectation based on a probit-transformation of the one-tailed probability 0 < *P* < 1 that the observed value is lower than expected^69–71^:

3 $$P=\frac{\#(null < obs)+\frac{\#(null=obs)}{2}}{1000}.$$

The probit-transform on *P* is implemented by using *qnorm* in the R statistical software version 3.4.3 (2017-11-30). The probit-transform accounts for skewness in data and is asymptotically equal to the standardised effect size introduced by^72^. We construct the average effect size by summing the effect sizes for each plot of a given richness.

We built our null models by assuming that growth or mortality are time-independent which would be the case if extreme climate events were not driving inter-annual variations in growth or mortality. For the growth model, we sampled from replacement a pool of BAGR from surviving trees in a given plot *over all years*. In this model, mortality is allowed to vary year on year. For the mortality model, mortality is similarly assumed *to be constant over the period of our analysis*. The instantaneous mortality rate *m* for each plot is then computed from ${N}_{t}={N}_{t-\Delta t}{e}^{-Tm}$ where *N*_*t*_ is the number of surviving trees at time *t* and *T* is the number of years our analysis extends over. Growth is allowed to vary year on year. The estimated 95% confidence interval is given by [−2.0, 2.0]^70^. We will thus consider that AES larger than 2.0 units indicates represents a significant effect of climate on growth/mortality at a given diversity level. AES that lie within the above interval are not significantly different from each other. We say that there is a biodiversity effect if differences between monocultures and mixtures manifest.

### Indicators of stability and slowing down

We use the averaged standardised effect size as an indicator of stability to climate extremes. The larger the effect size the less stable the system is to a perturbation due to drought or heavy rainfall. In the growth model, if the effect size is larger than 2.0 then growth is larger than expected by the null model and an effect size is less than −2.0 indicates less growth than expected. In the mortality model, if the effect size is larger than 2.0 then mortality is lower than expected and if the effect size is less than −2.0, mortality is greater than expected.

Temporal autocorrelation at lag-1 (TAC) can be measured in two equivalent ways^43^. The first way is to use

4 $${\rho }_{1}=\frac{{\rm{E}}[({z}_{t}-\mu )({z}_{t+1}-\mu )]}{{\sigma }_{z}^{2}}$$

where *μ* and *σ*_*z*_ are the mean and variance of the variable *z*_*t*_. In our case *z*_*t*_ is the basal area increment over year *t*. Alternatively, an autoregressive model of order 1, a linear AR(1)-process, of the form

5 $${z}_{t+1}={\alpha }_{1}{z}_{t}+{\varepsilon }_{t}$$

where *ε*_*t*_ represents Gaussian white noise. Here *ρ*_1_ and the autoregressive coefficient *α*_1_ are equivalent mathematically. We plot TAC against SPEI to determine whether there is an increase in autocorrelation with decreasing SPEI (i.e. more extreme drought conditions). We fit a single cubic spline to the data^42^ and construct confidence bands. For polynomial regression, the confidence band is constructed in the same way as multiple linear regression because the standard deviation of the estimators of the regression coefficients has the same expression in terms of the model matrix.

We compute the power spectrum using the R-function spec.pgrm (https://stat.ethz.ch/R-manual/R-devel/library/stats/html/spec.pgram.html) and we normalise the spectral density to be between zero and one.

## Electronic supplementary material

Supplementary Information

## Data Availability

The datasets generated during and/or analysed during the current study are available from the corresponding author on reasonable request.
