# Author Correction: Effect of diversity on growth, mortality, and loss of resilience to extreme climate events in a tropical planted forest experiment

**DOI:** 10.1038/s41598-019-53618-z

**Published:** 2019-11-22

**Authors:** Chantal Hutchison, Dominique Gravel, Frédéric Guichard, Catherine Potvin

**Affiliations:** 1 Department of Biology, McGill University, Montreal, H3A 1B1 Canada; 2 Département de biologie, Université de Sherbrooke, Sherbrooke, J1K 2R1 Canada

Correction to: *Scientific Reports* 10.1038/s41598-018-33670-x, published online 18 October 2018

An error was made in the computation of the SPEI value for 2016. It was reported as being an extreme dry year when it was, in fact, moderately wet. As a result, the original version of this Article contained minor inaccuracies in some of the text and figures. Fixing the error strengthens the main conclusions of the paper. A summary of these changes is given below.

In the Abstract,

“In contrast to higher richness mixtures, in one-species plots we find growth is strongly regulated by climate events and we also find increasingly higher mortality during a consecutive four year dry event.”

now reads:

“In contrast to higher richness mixtures, in one-species plots we find growth is strongly regulated by climate events and we also find increasingly higher mortality during a consecutive three year dry event.”

In the Results section, under the subheading ‘Climate events identified from the SPEI’,

“Based on a 21 year period from 1995 to 2016, we identify extreme climate events occurring approximately 20% of the time, moderate dry and wet events each occurring 25% of the time, and normal conditions persisting 30%.”

now reads:

“Based on a 21 year period from 1995 to 2016, we identify extreme climate events occurring approximately 10% of the time, moderate dry and wet events each occurring 25-30% of the time, and normal conditions persisting 35%.”

“Subsequently there is a series of dry years: 2013 and 2014 are moderately dry, and in 2015 and 2016 there are strong El Niño years with very dry conditions (Fig. 2).”

now reads:

“Subsequently there is a series of dry years: 2013 and 2014 are moderately dry, and in 2015 there is a strong El Niño year with very dry conditions (Fig. 2).”

In the Results section, under the subheading ‘Growth and mortality models’,

“During the drought episodes, which last from 2013 through to 2016, we see that although all mixtures experience a decrease in growth, monocultures experience the largest decrease and five-species mixtures the smallest decrease (Fig. 3a). In the mortality model, we see that the number of dead trees is pushed further from the null expectation for monocultures during the continuous drought period from 2013 to 2016 (Fig. 3b).”

now reads:

“During the drought episode from 2013 through to 2015, we see that although all mixtures experience a decrease in growth, monocultures experience the largest decrease and five-species mixtures the smallest decrease (Fig. 3a). In the mortality model, we see that the number of dead trees is pushed further from the null expectation for monocultures during the continuous drought period from 2013 to 2015 (Fig. 3b).”

In the Discussion section,

“We found that during the successive dry years from 2013 to 2016 there was increasingly higher mortality in monocultures compared to mixtures, which suggests that intraspecific competition is greater than interspecific competition during extreme climate events.”

now reads:

“We found that during the successive dry years from 2013 to 2015 there was increasingly higher mortality in monocultures compared to mixtures, which suggests that intraspecific competition is greater than interspecific competition during extreme climate events.”

“On the other hand, monocultures may simply be mirroring the environment. For example, we found that the spectral densities for monocultures closely follow that of the SPEI especially during dry years.”

now reads:

“In particular, the comparison of the spectral density of monocultures to SPEI suggests that they are not simply mirroring the environment.”

“We found that monocultures grow less than mixtures during a dry period from 2013 to 2016, culminating in the period from 2015 and 2016.”

now reads:

“We found that monocultures grow less than mixtures during a dry period from 2013 to 2015, culminating in 2015.”

“Mortality in five-species mixtures is significantly different from mixtures with intermediate levels of species richness during the drought period from 2013 to 2016, although both experienced less mortality than monocultures.”

now reads:

“Mortality in five-species mixtures is significantly different from mixtures with intermediate levels of species richness during the drought period from 2013 to 2015, although both experienced less mortality than monocultures.”

Figures 2, 3 and 4 have been replaced in the Article with the correct versions; the original versions are shown below as Figures 1, 2 and 3.

**Figure 1:**
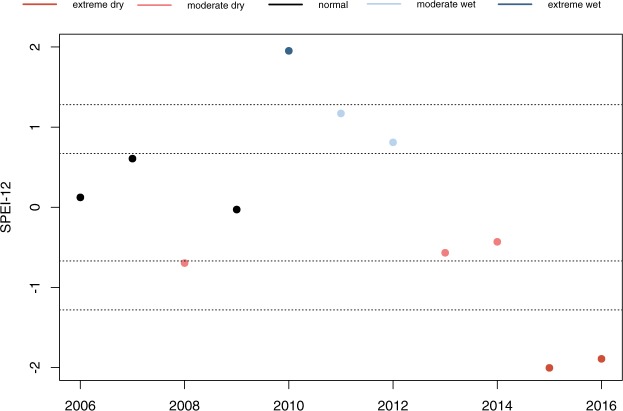
Original version of Figure 2, which is now replaced in the Article.

**Figure 2:**
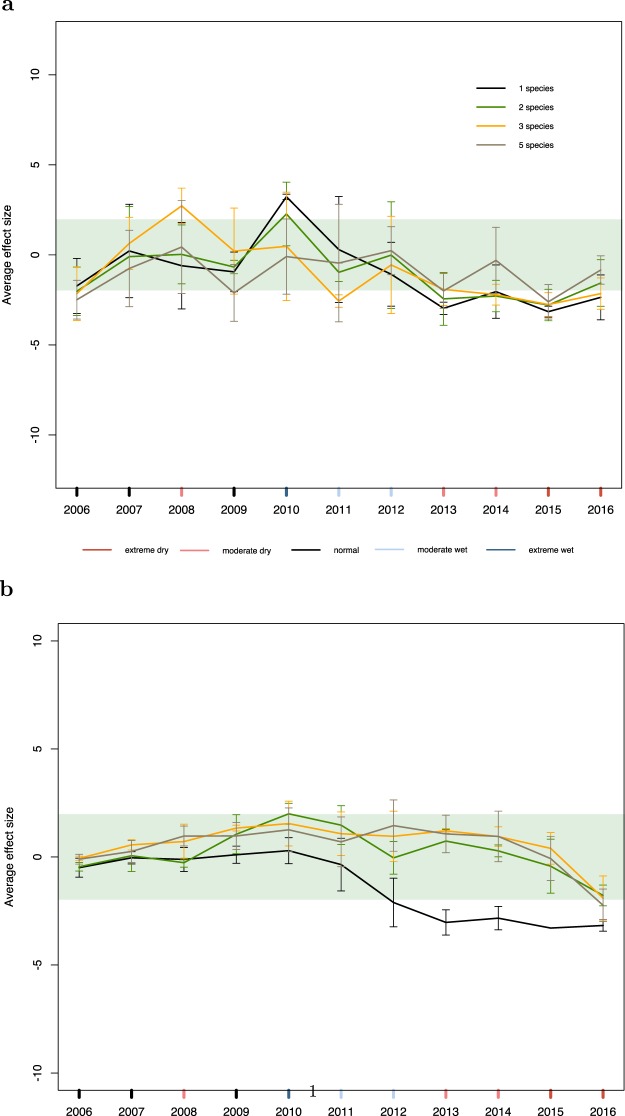
Original version of Figure 3, which is now replaced in the Article.

**Figure 3:**
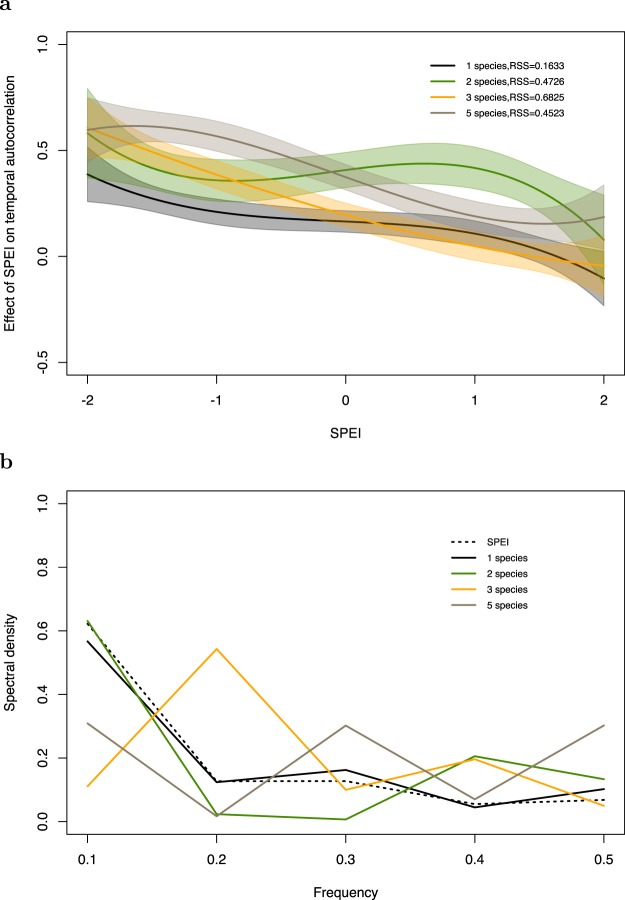
Original version of Figure 4, which is now replaced in the Article.

The legend of Figure 2,

“SPEI-12 (SPEI aggregated over twelve months) from December over 2006–2016. There is an extreme wet event in 2010 and an extreme dry spell from 2013 to 2016.”

now reads:

“SPEI-12 (SPEI aggregated over twelve months) from December over 2006-2012. There is an extreme wet event in 2010 and a dry spell from 2013 to 2015.”

