# Supplementary material for: Effect of diversity on growth, mortality, and loss of resilience to extreme climate events in a tropical planted forest experiment
Source: Sci Rep. 2018 Oct 18;8:15443. doi: 10.1038/s41598-018-33670-x (PMC6193960; doi:10.1038/s41598-018-33670-x)
Supplement: Supplementary file 1 — Supplementary Information [file 41598_2018_33670_MOESM1_ESM.pdf]

Supplementary information:
Effect of diversity on growth, mortality, and loss of resilience to extreme climate events in a tropical planted forest experiment

Chantal Hutchison, Dominique Gravel, Frédéric Guichard, Catherine Potvin

Figure S1

a

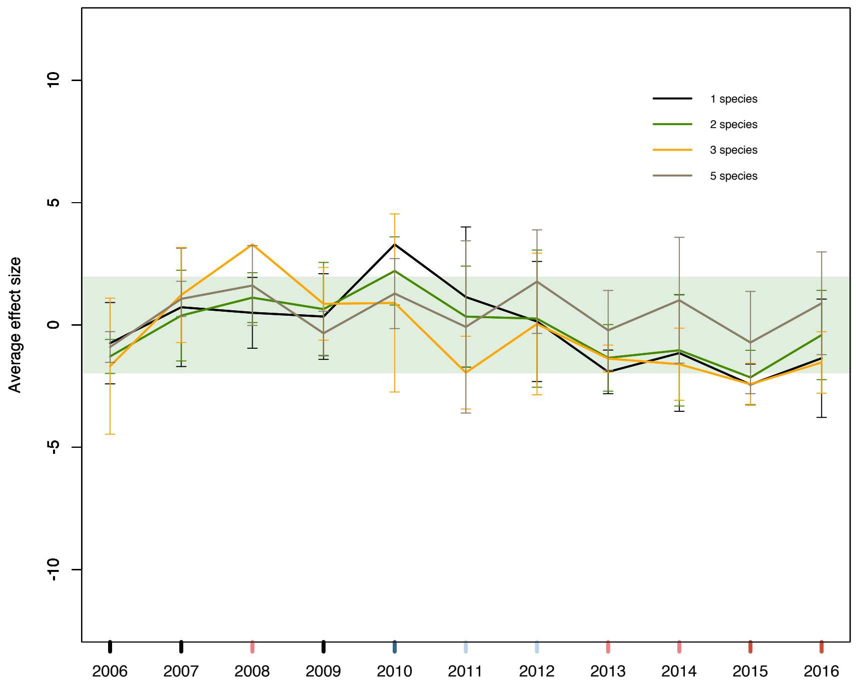

b

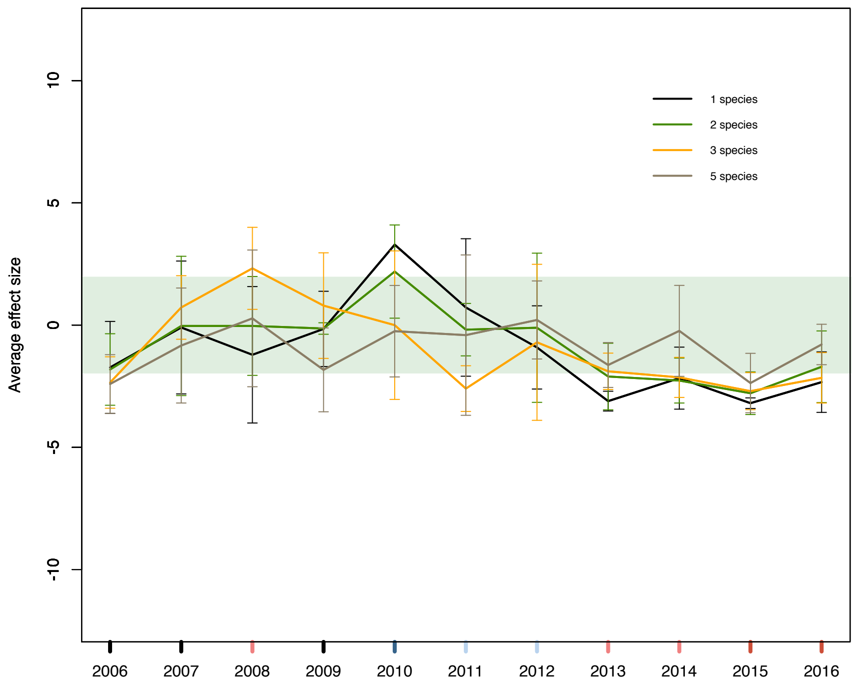

Sensitivity analysis including only (**a**) normal years (2006, 2007, and 2009) and (**b**) non-adjacent years in the pool for the growth model compared to all years as shown in the main text (Fig. 4). We find that our results are robust to changes in case **b**. The effect of the consecutive dry spell from 2013-2016 is absent in case **a**. One possibility is that growth which occurs in the three normal SPEI years of our study is not representative of the growth of the planted forest. It suggests that our choice of including all years in the pool for the growth model is conservative and unbiased.
